# The Polymorphism −308G/A of* Tumor Necrosis Factor-α* Gene Modulates the Effect of Immunosuppressive Treatment in First Kidney Transplant Subjects Who Suffer an Acute Rejection

**DOI:** 10.1155/2016/2197595

**Published:** 2016-09-29

**Authors:** Ana Isabel Sánchez-Fructuoso, Isabel Pérez-Flores, Rosalia Valero, Maria Angeles Moreno, Miguel Fernandez-Arquero, Elena Urcelay, Cristina Fernández-Pérez, Jose Luis Santiago

**Affiliations:** ^1^Nephrology Department, Hospital Clínico San Carlos, Instituto de Investigación Sanitaria San Carlos (IdISSC), Madrid, Spain; ^2^Immunology Department, Hospital Clínico San Carlos, Instituto de Investigación Sanitaria San Carlos (IdISSC), Madrid, Spain; ^3^Clinical Research and Methodology Unit, Hospital Clínico San Carlos, Instituto de Investigación Sanitaria San Carlos (IdISSC), Madrid, Spain

## Abstract

The −308G/A SNP of* tumor necrosis factor-alpha (TNF-α) gene* affects TNF-*α* production. As its impact on transplant outcome remains open to debate, we decided to genotype it in a cohort of transplant subjects. A retrospective analysis of 439 first kidney recipients randomly divided into two subgroups (discovery and validation cohorts) was performed to identify the best predictors of acute rejection (AR). The effect on transplant outcome was analyzed by an adjusted logistic regression model. Carriers of the A allele, associated with elevated TNF-*α* production, presented a higher risk of AR (OR = 2.78; 95% CI = 1.40–5.51). Logistic regression analyses for AR showed an interaction between the polymorphism and treatment with thymoglobulin (p-interaction = 0.03). In recipients who did not receive thymoglobulin, carriers of A allele had higher risk of AR (OR = 4.05; 95% CI = 1.76–9.28). Moreover, carriers of A allele not treated with thymoglobulin presented higher risk of AR than those who received thymoglobulin (OR = 13.74; 95% CI = 1.59–118.7). The AUC of the model in the discovery cohort was 0.70 and in the validation cohort was 0.69. Our findings indicate that the −308G/A* TNF-α* polymorphism is associated with AR risk and it modulates the effectiveness of thymoglobulin treatment. This pharmacogenetic effect lets us propose this SNP as a useful predictor biomarker to tailor immunosuppressive regimens.

## 1. Introduction

Acute rejection after kidney transplantation is a major cause of allograft dysfunction and can lead to rapid loss of graft function despite antirejection therapy. Even after initial recovery of kidney function, acute rejection is associated with an increased risk of long-term graft failure [1]. The identification of variables that can trigger rejection or modulate its severity could enable us to improve long-term allograft survival. The variables identified to date include younger age and African American ethnicity in the recipient, older donor age, the degree of donor-recipient human leukocyte antigen (HLA) mismatch, pretransplant anti-HLA alloantibodies, panel-reactive antibodies, ischemia-reperfusion injury (e.g., manifested by delayed graft function), and the adequacy of baseline immunosuppression [2]. It remains unknown why variations in the incidence of acute rejection are observed in patients with similar matching status who have received identical immunosuppressive protocols [3]. Much evidence exists to support the role of cytokines in the inflammatory and immune responses that mediate allograft rejection [4–6]. Tumor necrosis factor-alpha (TNF-*α*) is a proinflammatory cytokine produced by monocytes/macrophages and, to a lesser extent, by T cells and B cells [7, 8]. TNF-*α* is released at the site of inflammation, where it causes endothelial cell activation, upregulation of cell adhesion molecules and MHC expression, and increased vasodilatation and vascular permeability [9]. Therefore, TNF-*α* helps to maintain the inflammatory response to the allograft by facilitating recruitment and activation of leukocytes.

Many researchers have reported increased serum concentrations of TNF-*α* during acute rejection of liver [10], heart [11], and kidney [12, 13] allografts. The impact of TNF-*α* has been also reported in human renal allograft biopsies and in rat models of acute and chronic rejection [14, 15].

On the other hand, polymorphism in cytokine genes could explain differences in cytokine production and, therefore, in severity of rejection between individuals [16–18]. Polymorphism associated with cytokine production has been described in the gene encoding TNF-*α* [19]. The TNF-*α* gene is located in HLA class III region of the major histocompatibility complex (MHC) on chromosome 6p21.3 [15]. G-to-A single-nucleotide polymorphism (SNP) at position −308 in the* TNF-α* promoter region results in two forms related to their production, carriers of allele A, and GG genotype [20]. The presence of allele A is associated with increased transcriptional activity [19, 21] and elevated TNF-*α* production [22].

As local TNF-*α* release promotes endothelial cell activation and intragraft leukocyte migration, increased production of TNF-*α* could trigger rejection [10, 23, 24]. Consequently, the role of TNF-*α* polymorphism in acute graft rejection has been studied. Nonetheless, findings for the association between TNF-*α* and rejection are inconsistent. Some authors report that kidney recipients with the high-producing TNF-*α* −308A allele are at greater risk of rejection [16, 18, 22, 24–31], whereas other authors found no association [32–38]. Given the apparently controversial results of the studies performed to date, we investigated the impact of this polymorphism in a large cohort of well-characterized kidney recipients and validated our findings in a second cohort.

## 2. Subjects and Methods

### 2.1. Patients and Data Collection

**Figure 1:**
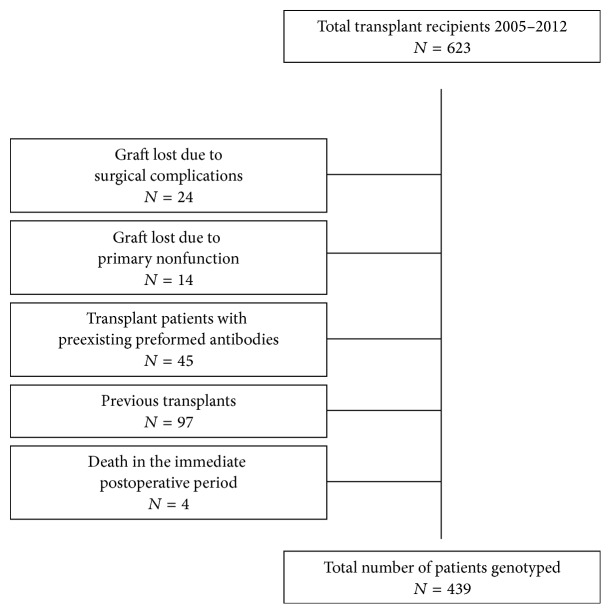
Flow-chart: kidney recipients.

Between January 2005 and December 2012, a total of 623 Caucasian adult patients (≥18 years) received a deceased donor organ and were followed up for at least 24 months in our center. We excluded 184 patients (Figure 1). The data recorded were as follows: demographic characteristics (recipient and donor), number of mismatches, immunosuppressive treatment, immediate or delayed graft function (need for dialysis in the first week after transplant), and type of donor (brain death or circulatory death). All diagnoses of rejection were confirmed by biopsy, and acute rejection was categorized according to the Banff classification [39]. Graft loss was defined as returning to chronic dialysis or death with a functioning graft. The clinical and research activities being reported are consistent with the Principles of the Declaration of Istanbul, as outlined in the Declaration of Istanbul on Organ Trafficking and Transplant Tourism. The protocol was approved by the Local Ethics Committee, and written informed consent was obtained from all patients.

### 2.2. Histopathology and C4d Staining

An ultrasound-guided graft biopsy was performed when clinically indicated, that is, in patients with elevated serum creatinine levels. All patients with delayed graft function underwent protocol biopsy every 7 days until kidney function began to improve. A representative biopsy involved at least 1 artery and more than 7 glomeruli. All Banff-scored lesions were assessed [39]. Deposition of C4d was studied by immunohistochemistry. Each patient with an acute rejection episode was tested for serum alloantibody. We classified acute rejection as follows: acute T cell rejection without vascular lesions, acute T cell rejection with vascular lesions, and antibody-mediated rejection according to the Banff classification [39, 40].

### 2.3. Immunosuppression

Patients who received a kidney from a brain dead donor were treated mainly with tacrolimus, mycophenolate mofetil, and methylprednisolone; when the donors had expanded criteria or when ischemia time was long, they also received basiliximab or thymoglobulin. When the organ was donated after circulatory death, most patients received treatment with tacrolimus, mycophenolate mofetil, and methylprednisolone combined with basiliximab or thymoglobulin. In patients who received thymoglobulin, tacrolimus was introduced between days 4 and 6 after transplant.

### 2.4. Cytokine Polymorphism Genotyping

Genomic DNA was extracted from EDTA-anticoagulated peripheral whole blood. The −308G/A* TNF-α* polymorphism (rs1800629) was genotyped in a 7900HT Fast Real-Time PCR System using a TaqMan assay (C_7514879_10, Applied Biosystems, Foster City, California, USA), as recommended by the manufacturer.

### 2.5. Statistical Analysis

Sample size was calculated based on an alpha risk of 0.05 and a beta risk of 0.2 in a 2-tailed contrast to detect a minimum relative risk of 2, assuming that 20% of patients not exposed to treatment would experience vascular rejection. It was calculated that 57 patients would be necessary in the high-producing group and 285 in the non-high-producing group (STATA, version 12.0). Kidney transplant recipients were randomly divided into two groups (2/3 and 1/3). The study of predictive factors was performed in 286 patients (discovery cohort) and subsequently validated in 153 patients (validation cohort). The influence of cytokine genotypes on acute rejection was expressed as a dichotomous variable, namely, low producers (GG) or high and intermediate producers (AA and GA). Qualitative variables were compared using the chi-square test or Fisher exact test and expressed as frequency distributions. Qualitative variables are expressed as mean (SD) or median (IQR) in the case of nonnormally distributed variables. They were compared using the *t*-test or nonparametric tests where necessary. An adjusted logistic regression model was constructed and included variables with *p* < 0.15 in the univariate analysis or variables that were biologically relevant in the population analysis. Interactions with TNF-*α* polymorphism were evaluated. The *p* value for the interaction was obtained from the models constructed. The adjusted odds ratios (Adj. OR) are presented with their 95% confidence intervals. Discriminatory power was evaluated using area under the receiver operating characteristic (ROC) curves (AUC) of the predicted probabilities obtained in the model. Calibration was assessed using the Hosmer-Lemeshow goodness-of-fit test in both the discovery cohort and the validation cohort. Survival of the kidney transplants as functioning organs was analyzed using the Kaplan-Meier method with a log-rank test. Null hypotheses with an alpha error <0.05 were rejected. The statistical package used was SPSS version 15.0.

## 3. Results

Of the total 439 patients in the cohort, 119 (27.1%) developed acute rejection (AR); of these, 83 experienced vascular involvement (18.9%). Median follow-up was 62.5 (39.9–87.2) months. The median time to rejection was 9 (7–15) days, and 96.6% of all rejections were during the first year after transplantation. The genotype distribution of −308A/G* TNF-α* was 82.9% (*N* = 364) for GG, 15.3% (*N* = 67) for GA, and 1.8% (*N* = 8) for AA.

The study of predictive factors was performed in 286 patients and subsequently validated in 153 patients. The distribution of risk factors was similar between the two cohorts (Table 1). The results of the univariate analysis for AR in the discovery cohort are shown in Table 2. The variables significantly associated with a greater risk were age (donor and recipient), the −308A/G polymorphism, and the immunosuppressive treatment. Carriers of the A allele had a greater risk of AR than patients with the GG genotype (Table 2).

**Figure 2:**
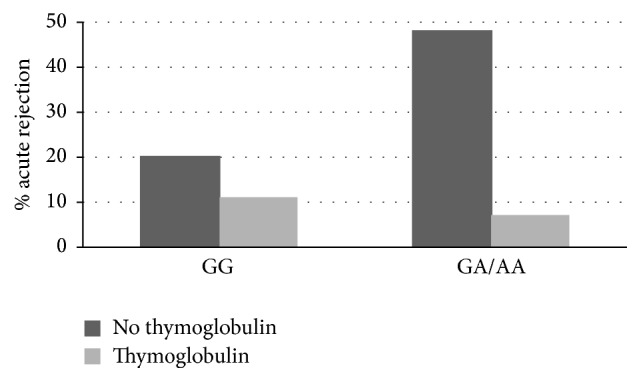
Percentage of AR in renal transplant patients stratified both −308G/A* TNF-α* gene polymorphism and treatment with thymoglobulin.

The model was adjusted to evaluate the development of acute cellular rejection and only the statistically significant variables were shown (Table 3). In this multivariate analysis, a significant interaction was recorded between induction treatment with thymoglobulin and the polymorphism in* TNF-α* (*p* = 0.03) (Table 3). Carriers of the A allele who were not treated with thymoglobulin had a 4.05 times greater risk of vascular rejection than those harboring GG (Table 3) (Figure 2). Furthermore, in the subgroup of carriers of A allele, the probability of rejection was considerably higher (13-fold) in patients not receiving thymoglobulin than in those receiving thymoglobulin (Table 3). The area under the ROC curve of the model in the discovery cohort was 0.70 (95% CI = 0.62–0.78); the *p* value of the Hosmer-Lemeshow test was 0.916. The model was subsequently applied in the validation cohort, and the area under the ROC curve was 0.69 (95% CI = 0.54–0.76).

## 4. Discussion

Alloimmune responses and differences in susceptibility to rejection may be influenced by individual variations in cytokine genes. Indeed, cytokine gene polymorphism types have been extensively explored in transplantation because they are thought to explain the heterogeneous outcomes of the allograft and can thus help clinicians to tailor immunosuppression [5, 31, 33, 41–43].

Several studies have assessed the association between the −308A/G TNF-*α* polymorphism and acute rejection in kidney recipients of different populations; however, the results are apparently inconsistent and inconclusive. Our data support reports that found association of this SNP with a higher incidence of acute rejection [16, 18, 22, 24–31]. In their meta-analysis, Hu et al. [44] concluded that the TNF-*α* high producers genotypes in the recipient were associated with an increased risk of acute allograft rejection. In this meta-analysis, authors recommended performing additional studies with large sample size and better study designs.

Discrepancies in previously reported findings may be due to a small size that compromised the statistical power and heterogeneity of the studies. Only few reports included cohorts of consecutive transplants performed over a specific time period [16, 24, 33, 34, 37] and some even recruited hyperimmunized and retransplanted patients [16, 26, 30, 31, 33, 34, 37]. Moreover, rejection was not histologically confirmed in most studies and incidence of rejection also varies considerably, ranging from 17% [16] to 63% [24]. In addition, discrepancies in terms of the impact of the TNF-*α* polymorphism in kidney rejection could also arise because of differences in the immunosuppressive therapy used, as the type of medication administered is not usually included. The most common immunosuppressive agents are cyclosporine, prednisone, and azathioprine or mycophenolate [16, 18, 24–27, 29–34, 36–38]. Our results suggest that use of triple therapy based on tacrolimus, mycophenolate, and corticosteroids may not be sufficient to block release of TNF-*α* in patients with the high producer genotype. TNF-*α* is mainly generated by monocytes and macrophages [45] and it has been reported that the aforementioned triple therapy does not have a clear effect in these cells [46–48]. However, antithymocyte globulin promotes expansion of regulatory T cells [49], the main producers of interleukin-10, which in turn inhibits production of TNF-*α* by macrophages [50]. Therefore, antithymocyte globulin could help to control the immune response in patients who produce high levels of TNF-*α*.

Our study is limited by the fact that TNF-*α* genotyping was not performed in the donor. Nevertheless, bearing in mind that most TNF-*α* is produced by macrophages, we think that more emphasis should be placed on the receptor genotype.

Before a model can be relied upon to draw conclusions or predict future outcomes, it is important to ensure that it is correctly specified; that is, the data do not conflict with assumptions made by the model. Logistic regression is the most popular modeling approach for binary outcomes. The Hosmer-Lemeshow test makes it possible to compare goodness of fit by comparing observed and predicted risks across subgroups in a population. Prediction models allow clinicians to estimate prognosis [51, 52] and are increasingly used in clinical practice to guide decision-making [52]. Our study is the first one that retrospectively analyzed a prospective cohort of first transplants in patients with no preformed antibodies who were randomly assigned to two cohorts, a discovery and a validation cohort. We attempted to predict the risk of acute rejection according to variables that can modify the effect simultaneously (age, delayed graft failure, immunosuppression protocol, and the TNF-*α* polymorphism).

## 5. Conclusions

The* TNF-α gene* polymorphism that was previously associated with differential production of this cytokine is associated with AR risk and modulates the effectiveness of thymoglobulin treatment. Screening of this polymorphism will enable us to predict those patients (carriers of A allele) more likely to experience rejection and, therefore, require more intense immunosuppressive therapy. Similarly, it will enable us to identify patients with a potentially optimal response, who can be treated with less potent immunosuppression.

## Figures and Tables

**Table 1:** Characteristics of the two randomly divided cohorts of kidney recipients.

	Discovery cohort (*n* = 286)	Validation cohort (*n* = 153)	*p* value
Recipient age, years, mean ± SD	52.2 ± 13.5	51.8 ± 13.0	0.78
Male recipient, *N* (%)	191 (66.8%)	100 (65.4%)	0.76
Time on dialysis, months	17.8 (7.1–31.2)	16.8 (6.1–26.9)	0.58
Cause of chronic renal failure, *N* (%)			0.08
Glomerulonephritis	86 (30.1%)	53 (34.6%)	
Chronic tubulointerstitial nephropathy	31 (10.8%)	23 (15.0%)	
Nephroangiosclerosis	22 (7.7%)	11 (7.2%)	
Polycystic kidney disease	45 (15.3%)	21 (13.7%)	
Diabetic nephropathy	42 (14.7%)	8 (5.2%)	
Unknown cause	48 (16.8%)	32 (20.9%)	
Others	12 (4.2%)	5 (3.3%)	
Donor age, years, mean ± SD	43.0 ± 14.3	42.6 ± 14.1	0.73
Male donor, *N* (%)	209 (73.6%)	112 (74.2%)	0.90
Donor type, *N* (%)			0.65
Brain death	111 (38.8%)	56 (36.6%)	
Circulatory death	175 (61.2%)	97 (61.2%)	
Immunosuppressive treatment, *N* (%)			0.87
Thymoglobulin + FK + MMF + P	98 (34.3%)	54 (35.3%)	
IL2R + FK + MMF + P	127 (44.4%)	69 (45.1%)	
FK + MMF + P	54 (18.9%)	28 (18.3%)	
CsA + MMF + P	2 (0.7%)	0 (0)	
FK + SRL + P	1 (0.3%)	1 (0.7%)	
Belatacept + MMF + P	4 (1.4%)	1 (0.7%)	
Follow-up time, months (median [IQR])	74.9 (53.8–99.5)	73.0 (50.2–97.5)	0.54
Delayed graft function, *N* (%)	143 (50.0%)	80 (52.3%)	0.65
HLA-A mismatch, *N* (%)			0.56
0	24 (8.4%)	17 (11.3%)	
1	118 (41.4%)	64 (42.4%)	
2	143 (50.2%)	70 (46.4 %)	
HLA-B mismatch, *N* (%)			0.55
0	12 (4.2%)	4 (2.6%)	
1	112 (39.3%)	66 (43.4%)	
2	161 (56.5%)	82 (53.9%)	
HLA-DR mismatch, *N* (%)			0.13
0	34 (11.9%)	18 (11.8%)	
1	123 (43.2%)	80 (52.6%)	
2	128 (44.9%)	54 (35.5%)	
Acute total rejection, *N* (%)	80 (28.0%)	39 (25.5%)	0.58
Acute rejection Banff ≥ 2, *N* (%)	55 (19.2%)	28 (18.3%)	0.81
Acute humoral rejection, *N* (%)	20 (7.0%)	10 (6.5%)	0.86
Genotype frequency GA/AA TNF-*α* −308, *N* (%)	49 (17.1%)	26 (17.0%)	0.97
Graft loss, *N* (%)	49 (17.1%)	21 (13.7%)	0.35

FK: tacrolimus; MMF: mycophenolate; P: prednisone; IL2R: interleukin- (IL-) 2 receptor antagonist; CsA: cyclosporin A; SRL: sirolimus.

**Table 2:** Univariate analysis for acute rejection in the discovery cohort (*n* = 286).

	Acute rejection *N* (%)	OR (95% CI)	*p* value
Recipient age			0.04
<60 years	43 (22.6%)	2.04 (1.02 to 4.17)	
≥60	12 (12.5%)	1	
Recipient sex			0.17
Male	41 (21.5%)	1.59 (0.81 to 3.03)	
Female	14 (14.7%)	1	
Donor age			0.05
<60 years	54 (20.6%)	5.88 (0.81 to 50.0)	
≥60	1 (4.2%)	1	
Donor sex			0.22
Male	44 (21.1%)	1.72 (0.83 to 3.70)	
Female	11 (14.5%)	1	
Time on dialysis			0.64
<15 months	24 (18.0%)	1	
≥15 months	31 (20.3%)	1.15 (0.64 to 2.09)	
Donor type			0.47
Brain death	19 (17.1%)	1	
Circulatory death	36 (20.6%)	1.25 (0.68 to 2.32)	
Delayed graft function			0.45
Yes	30 (21.0%)	1.25 (0.70 to 2.26)	
No	25 (17.5%)	1	
TNF-*α* −308 polymorphism			0.003
GG	38 (16.0%)	1	
GA/AA	17 (34.6%)	2.78 (1.40 to 5.51)	
Immunosuppressive treatment			0.003
Thymoglobulin + FK + MMF + P	8 (8.2%)	1	
IL2R + FK + MMF + P	32 (25.2%)	3.74 (1.63 to 8.57)	
Other (belatacept, SRL)	15 (24.2%)	3.55 (1.40 to 8.98)	
HLA mismatch			0.75
<3	4 (22.2%)	1.20 (1.38 to 3.85)	
≥3	51 (19.1%)	1	
HLA-DR mismatch			0.22
≤1	34 (21.9%)	1	
2	21 (16.2%)	0.69 (0.38 to 1.25)	
HLA-A mismatch			0.86
≤1	28 (19.7%)	1	
2	27 (18.9%)	0.95 (0.52 to 1.71)	
HLA-B mismatch			0.56
≤1	22 (17.7%)	1	
2	33 (20.5%)	1.20 (0.66 to 2.18)	

FK: tacrolimus; MMF: mycophenolate; P: prednisone; IL2R: interleukin- (IL-) 2 receptor antagonist; SRL: sirolimus.

**Table 3:** Multivariate analysis for acute rejection in the discovery cohort (*n* = 286).

Variable	OR (95% CI)^*∗*^	*p* value
No thymoglobulin treatment		
TNF-*α* −308 GG	1	
TNF-*α* −308 GA/AA	4.05 (1.76 to 9.28)	0.001
Thymoglobulin treatment		
TNF-*α* −308 GG	1	
TNF-*α* −308 GA/AA	0.65 (0.12 to 3.69)	0.65
TNF-*α* −308 GG		
Thymoglobulin treatment	1	
No thymoglobulin treatment	2.72 (1.05 to 7.05)	0.04
TNF-*α* −308 GA/AA		
Thymoglobulin treatment	1	
No thymoglobulin treatment	13.74 (1.59 to 118.7)	0.02
Recipient age		
≥60 years	1	
<60 years	2.29 (1.10 to 4.78)	0.03

^*∗*^Adjusted for recipient sex, donor age and sex, HLA-DR mismatches, and delayed graft function.

p-interaction (thymoglobulin treatment and TNF-*α*  −308 polymorphism) = 0.03.
